# A hierarchy of corticospinal plasticity in human hand and forearm muscles

**DOI:** 10.1113/JP277462

**Published:** 2019-03-20

**Authors:** K. M. Riashad Foysal, Stuart N. Baker

**Affiliations:** ^1^ Institute of Neurosciences Newcastle University Newcastle upon Tyne UK

**Keywords:** Plasticity, Transcranial magnetic stimulation, Motor cortex, Corticospinal tract

## Abstract

**Key points:**

- Pairing stimulation of a finger flexor or extensor muscle at the motor point with transcranial magnetic stimulation (TMS) of the motor cortex generated plastic changes in motor output.
- Increases in output were greater in intrinsic hand muscles than in the finger flexor. No changes occurred in the finger extensor. This gradient was seen irrespective of which muscle was stimulated paired with transcranial magnetic stimulation.
- Intermittent theta‐burst stimulation also produced increases in output, although these were similar across muscles.
- We suggest that intrinsic hand and flexor muscles have a higher potential to show plasticity than extensors, although only when plasticity is induced by sensory input. This may relate to differences seen in recovery of function in these muscles after injury, such as post‐stroke.

**Abstract:**

The ability of the motor system to show plastic change underlies skill learning and also permits recovery after injury. One puzzling observation is that, after stroke, upper limb flexor muscles show good recovery but extensors remain weak, with this being a major contributor to residual disability. We hypothesized that there might be differences in potential for plasticity across hand and forearm muscles. In the present study, we investigated this using two protocols based on transcranial magnetic brain stimulation (TMS) in healthy human subjects. Baseline TMS responses were recorded from two intrinsic hand muscles: flexor digitorum superficialis (FDS) and extensor digitorum communis (EDC). In the first study, paired associative stimulation (PAS) was delivered by pairing motor point stimulation of FDS or EDC with TMS. Responses were then remeasured. Increases were greatest in the hand muscles, smaller in FDS and non‐significant in EDC, irrespective of whether stimulation of FDS or EDC was used. In the second study, intermittent theta‐burst rapid rate TMS was applied instead of PAS. In this case, all muscles showed similar increases in TMS responses. We conclude that the potential to show plastic changes in motor cortical output has the gradient: hand muscles > flexors > extensors. However, this was only seen in a protocol that requires integration of sensory input (PAS) and not when plasticity was induced purely by cortical stimulation (rapid rate TMS). This observation may relate to why functional recovery tends to favour flexor and hand muscles over extensors.

## Key points

- Pairing stimulation of a finger flexor or extensor muscle at the motor point with transcranial magnetic stimulation (TMS) of the motor cortex generated plastic changes in motor output.
- Increases in output were greater in intrinsic hand muscles than in the finger flexor. No changes occurred in the finger extensor. This gradient was seen irrespective of which muscle was stimulated paired with transcranial magnetic stimulation.
- Intermittent theta‐burst stimulation also produced increases in output, although these were similar across muscles.
- We suggest that intrinsic hand and flexor muscles have a higher potential to show plasticity than extensors, although only when plasticity is induced by sensory input. This may relate to differences seen in recovery of function in these muscles after injury, such as post‐stroke.

## Introduction

Synaptic connections in the central nervous system are not fixed and can be modified on the basis of learning or behaviour in healthy individuals (Classen *et al*. 1998). Such plasticity assumes great importance during recovery after brain lesions, when it permits the strengthening of residual pathways to compensate for damage (Benecke *et al*. 1991; Baker *et al*. 2015). In monkeys after a corticospinal tract lesion, we previously demonstrated that inputs to motoneurons from the remaining reticulospinal tract strengthened to forearm flexor and intrinsic hand motoneurons (Zaaimi *et al*. 2012). Coupled with changes in activity in the reticular formation itself (Zaaimi *et al*. 2018), these changes could restore much of the lost corticospinal drive and thus explain why the flaccid paralysis seen immediately post‐lesion rapidly improves. However, reticulospinal inputs to forearm extensor muscles do not change (Zaaimi *et al*. 2012). This probably underlies one of the important residual disabilities in stroke survivors, who, despite regaining good grasp, have very weak finger and wrist extensors. Extensor weakness prevents hand opening, substantially degrading functional use of the hand (Kamper *et al*. 2003).

Why do connections to extensors show so little plasticity after injury? One possible explanation is that residual pathways strengthen according to their pre‐existing bias. The reticulospinal tract is known to preferentially facilitate ipsilateral flexors (Davidson & Buford, 2006) and so it might therefore appear unsurprising that outputs to flexors should be especially enhanced during recovery. However, the reticular formation preferentially facilitates contralateral extensors (Davidson & Buford, 2006). Because corticoreticular connections from a given hemisphere are bilateral (Fregosi *et al*. 2017), there is a clear route for corticoreticulospinal activation of extensors. Despite the existence of this pathway in health, it does not appear to be strengthened after lesion. The rubrospinal tract has a clear bias towards activating extensor muscles in healthy monkey (Mewes & Cheney, 1991). Nevertheless, after a corticospinal tract lesion, rubrospinal inputs also strengthen to flexor but not extensor motoneurons (Belhaj‐saif & Cheney, 2000; Zaaimi *et al*. 2012). In healthy animals, corticospinal connections to extensors may be stronger than to flexors (Fetz & Cheney, 1980), although this bias is weak (Park *et al*. 2004). A corticospinal lesion should thus leave at least as large a vacant synaptic territory on extensor compared to flexor motoneurons, providing a powerful stimulus for terminal sprouting and enhancement of connections from residual pathways (Brus‐Ramer *et al*. 2007; Tan *et al*. 2012). However, strengthening does not occur to extensors.

An alternative possibility is that there are innate differences in the ability to show plasticity in neural circuits controlling different muscle groups. This may relate to a different ability to control precise movements between flexors and extensors. For example, forehand tennis serves are more accurate than backhand serves (Mavvidis *et al*. 2010), finger flexion movements show increased individuation compared to extension (Schieber, 1991) and finger flexion forces are more precise than extension (Divekar & John, 2013). The molecular mechanisms required for synaptic plasticity are complex; from an evolutionary perspective, there might be no advantage in deploying these systems for muscle groups that are mostly called upon to make stereotyped movements.

In the present study, we compared plastic changes in corticospinal outputs to different human forearm and hand muscles induced by two non‐invasive stimulation protocols. Paired associative stimulation (PAS) involves pairing peripheral stimulation with transcranial magnetic stimulation (TMS) over the motor cortex, with an interstimulus interval chosen to allow convergence of the stimulus effects at the cortex (Stefan *et al*. 2000). We found that a modified PAS protocol induced larger plastic changes in intrinsic hand muscles than in forearm flexors and did not significant modify outputs to extensors at all. By contrast, repetitive TMS (rTMS) delivered as intermittent theta‐burst stimulation (iTBS) over the motor cortex generated similar changes in all muscle groups examined. The results support the hypothesis of underlying differences in plasticity between muscle groups, although these differences were only apparent using a protocol that required the integration of sensory input.

## Methods

### Subjects

The data obtained in the present study were recorded from 23 healthy volunteers (eight males, 15 females; age range 19–50 years) in 30 sessions for the PAS experiment and from nine healthy volunteers (four males, five females; age range 19–32 years) in 23 sessions for the rTMS experiments. Tests from six rTMS sessions could not be completed as a result of coil overheating and these were excluded from the analysis. All procedures were approved by the local ethical committee of Newcastle University Medical School (ethical approval number 000023/2008). Prior to each experiment, full written consent was obtained from each participant after explaining each procedure in detail. Subjects were seated in a comfortable, height adjustable chair during the study, with their right forearm resting on an adjacent table. They were instructed to stay relaxed during the entire experiment; this was ensured by continual monitoring of the electromyogram (EMG) recordings by the experimenter. Some subjects participated in more than one experiment, with an intervening interval of at least 7 days.

### Recordings

An EMG recording was made from the right flexor digitorum superficialis (FDS), extensor digitorum communis (EDC), abductor pollicis brevis (APB) and first dorsal interosseous (1DI), using adhesive surface electrodes (model H59P Kendall; Covidien, Dublin, Ireland) placed over the muscle belly (separation 2–3 cm for FDS/EDC, ∼1 cm for APB/1DI). Electrodes were connected to a model D360 amplifier (Digitimer Ltd, Welwyn Garden City, UK) (gain, 1000; bandpass filter, from 30 Hz to 2 kHz) and signals digitized (micro1401 laboratory interface; Cambridge Electronics Design, Cambridge, UK) and stored on a personal computer system (Spike2 software; Cambridge Electronic Design).

### TMS

For PAS experiments, a figure‐of‐eight shaped magnetic coil (model D70^2^) and Magstim 200^2^ stimulator (Magstim Ltd, Whitland, UK) delivered TMS to the left hemisphere. For the rTMS study, a similar coil was connected to a Magstim Rapid^2^ stimulator (Magstim Ltd). In both cases, the coil was positioned with the handle pointing posterior and lateral at 45° to the sagittal plane, leading to the initial induced current in the brain flowing in a posterior–anterior direction. Coils were fitted with optical markers, for which the position was tracked relative to similar markers placed on a forehead headband, allowing careful control of coil location (Brainsight; Rogue Resolutions Ltd, Cardiff, UK).

The TMS coil was moved over the head to locate the optimal location for motor‐evoked potentials (MEPs) from the FDS and EDC muscles; this was then marked as the hotspot using the Brainsight system, with all subsequent stimuli being delivered at this location. The resting motor threshold (RMT) was determined as the minimum intensity required to evoke >50 μV peak‐to‐peak responses in both relaxed muscles in at least five out of 10 trials. In both PAS and rTMS studies, MEP amplitudes were measured at 1.2 × RMT.

### PAS protocol

**Figure 1:**
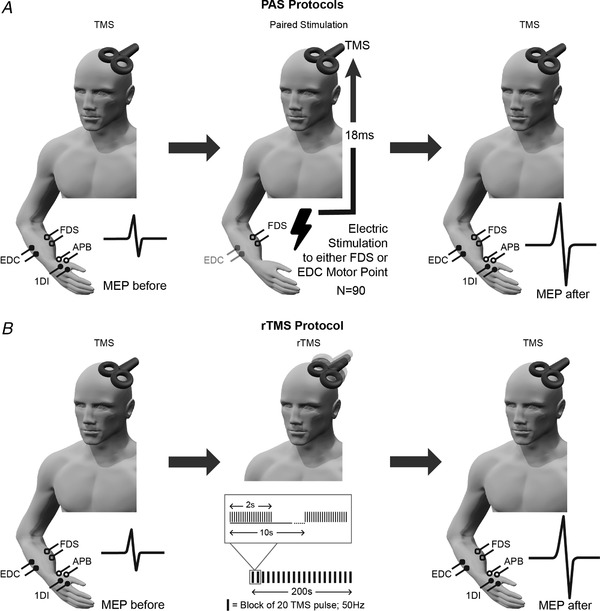
Experimental set‐up and protocols In all cases, MEPs were recorded from four muscles (APB, abductor pollicis brevis; 1DI, first dorsal interosseous; FDS, flexor digitorum superficialis; EDC, extensor digitorum communis) before and after an intervention intended to induce plasticity. *A*, PAS protocols; electrical stimulation was applied to a muscle motor point 18 ms before TMS. Either FDS or EDC muscle was stimulated, for a total of 90 paired stimuli. *B*, rTMS protocol; 20 pulses of TMS were delivered at frequency of 50 Hz as a block and each block was delivered at a frequency of 0.1 Hz for 190s.

The study began by recording baseline MEPs from all four muscles, using one or two sets of 20 magnetic stimuli at a frequency of 0.1 Hz (Fig. 1 *A*). PAS was then delivered by pairing electrical stimulation of either the FDS or EDC muscle (0.5 ms pulse width; 2–3 × motor threshold; stimuli delivered through the EMG electrodes using DS7A constant‐current isolated stimulator; Digitimer Ltd) with TMS (intensity 1.2 × RMT). Muscle stimulation was delivered 18 ms prior to the TMS. This interval was slightly lower than the 20–25 ms typically used for PAS when stimulating the median nerve at the wrist, aiming to compensate for the more proximal location of the stimulus site. In total, 90 paired stimuli were given at a frequency of 0.05 Hz (pairing duration of 30 min). Immediately after PAS, two sets of 20 MEPs were again recorded.

### rTMS protocol

A Rapid^2^ stimulator was used to measure the active motor threshold (AMT), defined as the minimum intensity required to evoke a MEP with 100 μV peak‐to‐peak amplitude in at least five trials out of 10 during a steady voluntary contraction (20% of maximal contraction) of 1DI. A Magstim 200^2^ stimulator was used to determine the RMT, as described above. We then recorded 30 MEPs using the Magstim 200^2^ stimulator at a frequency of 0.2 Hz and an intensity of 1.2 × RMT (Fig. 1 *B*). rTMS was delivered using a modified iTBS paradigm and the Magstim Rapid^2^ (Huang *et al*. 2005). Pairs of stimuli (intensity 0.9 × AMT) with 20 ms spacing were delivered every 200 ms for 2 s; such stimulus trains were given at a frequency of 0.1 Hz for 190 s, amounting to a total of 400 pulses during a single intervention session. Occasionally, the stimulus coil overheated, such that stimulation had to be briefly paused to allow the coil to cool. MEP measurements (*n* = 40 stimuli) were started again immediately following iTBS using the same stimulation parameters as at baseline.

### Statistical analysis

Averages of rectified EMG recordings were compiled and used to determine the MEP onset and offset latencies for each muscle separately. The area under the curve between these latencies was determined from individual sweeps and averaged to measure the MEP amplitude. Significant differences before and after the intervention were assessed by performing *t* tests on the single sweep values. Changes after the intervention were expressed as a percentage of the measurement before. An ANOVA with factors recorded muscle and (for PAS) stimulated muscle was performed to assess changes at the population level, with subsequent pairwise testing as required (*t* tests). *P* < 0.05 was considered statistically significant. All analyses were carried out using custom‐written scripts in the MATLAB environment (MathWorks Inc., Natick, MA, USA).

## Results

**Figure 2:**
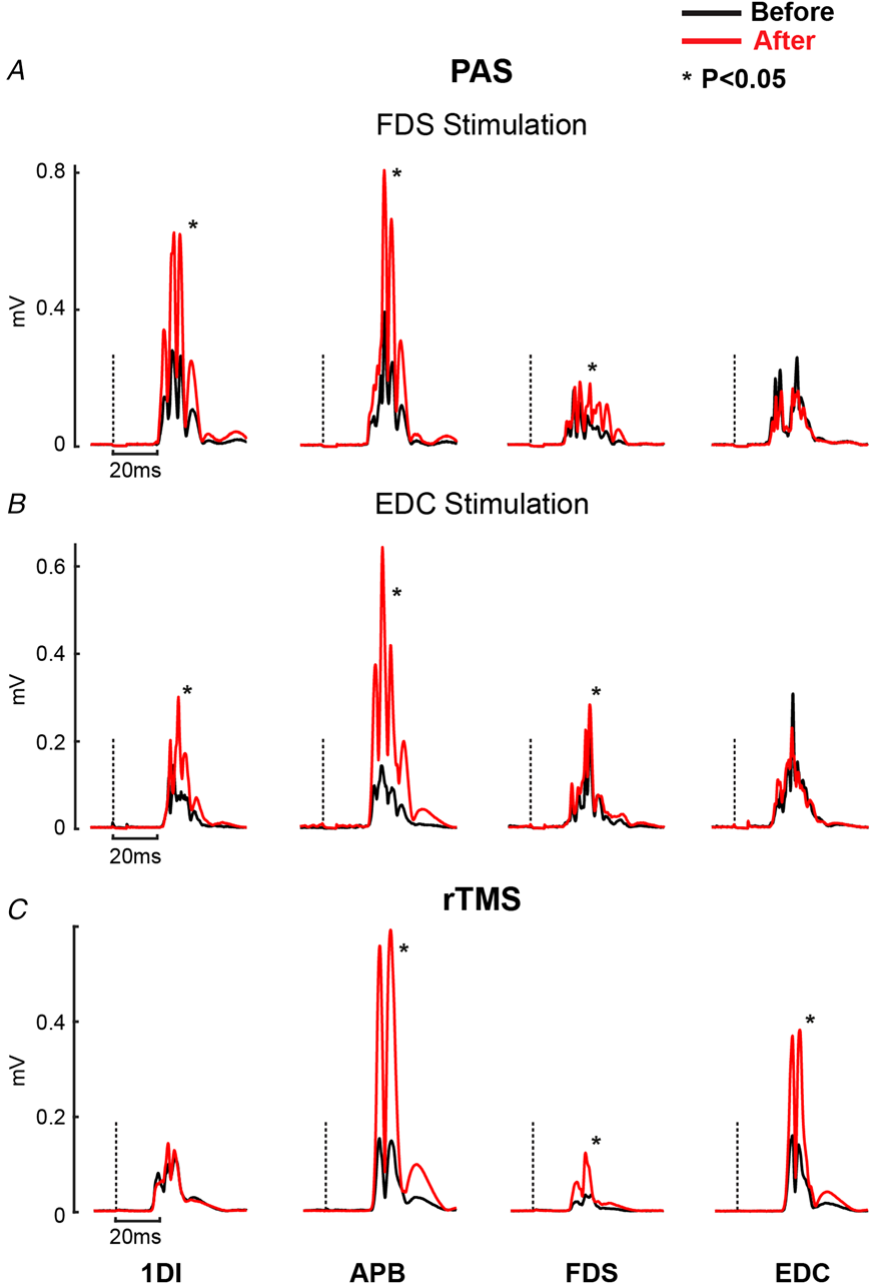
Example results from a single subject *A*, average rectified MEPs from 1DI, APB, FDS and EDC muscles before (black) and after (red) PAS protocol with FDS stimulation. *B*, as described in (*A*) but for a PAS experiment with EDC stimulation. *C*, as described in (*A*) but for the rTMS protocol. A dotted line indicates the time of TMS. ^*^Statistically significant increase in MEPs recorded after the intervention (*P* < 0.05, *t* test). [Color figure can be viewed at wileyonlinelibrary.com]

Figure 2 presents example results obtained from single subjects for each of the plasticity protocols tested. Following PAS using stimulation of the FDS motor point (Fig. 2 *A*), the MEP was significantly increased for the two intrinsic hand muscles and for FDS (increases after PAS as a percentage of control: 1DI, 129%; APB, 125%; FDS, 85%; all *P* < 0.0001). By contrast, the EDC muscle exhibited a small decline in MEP of 20% compared to baseline, which just reached significance (*P* < 0.042). This result might be expected: facilitation was seen in the stimulated muscle but not in its antagonist. However, surprisingly, when PAS paired TMS with stimulation over the EDC muscle, a very similar result was obtained (Fig. 2 *B*). Again, the responses in 1DI, APB and FDS were all significantly greater after paired stimulation (increases relative to baseline 97%, 254% and 49%, respectively; *P* < 0.005), although there was no significant change in the MEP from EDC (*P* > 0.05).

By contrast to PAS, rTMS appeared able to induce plastic changes also in the EDC muscle (Fig. 2 *C*). In this subject, the MEPs in the APB, FDS and EDC muscles were all significantly increased (increases relative to baseline of 223%, 131% and 121% respectively, all *P* < 0.05). The MEP in the 1DI muscle was not significantly changed (*P* > 0.05).

**Figure 3:**
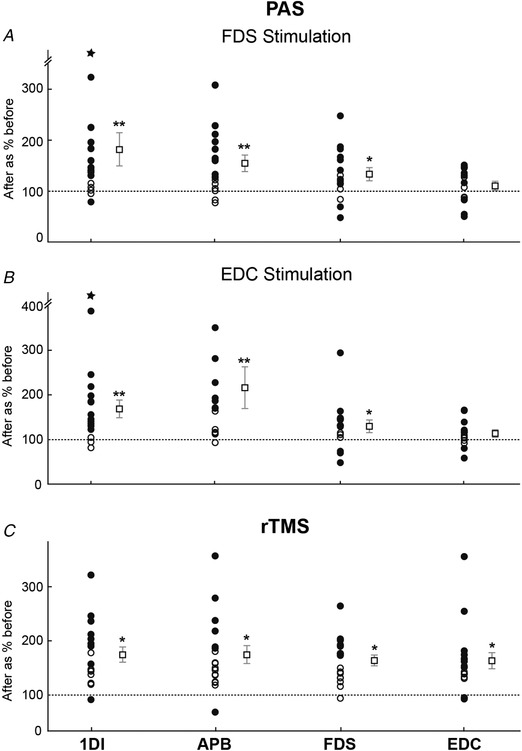
Group results Individual MEP changes are shown as circles, calculated as the size of the MEP after intervention as a percentage of the MEP before intervention. Filled circles denote subjects with a statistically significant difference (*P* < 0.05, *t* test). To the right of each set of data for a single subject, squares are plotted showing the mean ± SEM across the population; asterisks indicate statistically significant increases (^*^ *P* < 0.05; ^**^ *P* < 0.005, *t* test). *A*, PAS with FDS stimulation. *B*, PAS with EDC stimulation. *C*, rTMS.

Figure 3 presents group results across all subjects, with the MEP amplitude after the intervention being expressed as a percentage of the baseline measure (individual data points with significant changes are shown as filled circles; group means are shown to the right of the individual data points). Both PAS protocols yielded significant changes in 1DI, APB and FDS; however, in the EDC muscle, MEPs were not significantly altered after PAS, irrespective of whether stimulation of the EDC or FDS muscle was paired with TMS (Fig. 3 *A*, *B*). By contrast, rTMS produced significant increases in responses for all four muscles (Fig. 3 *C*).

**Figure 4:**
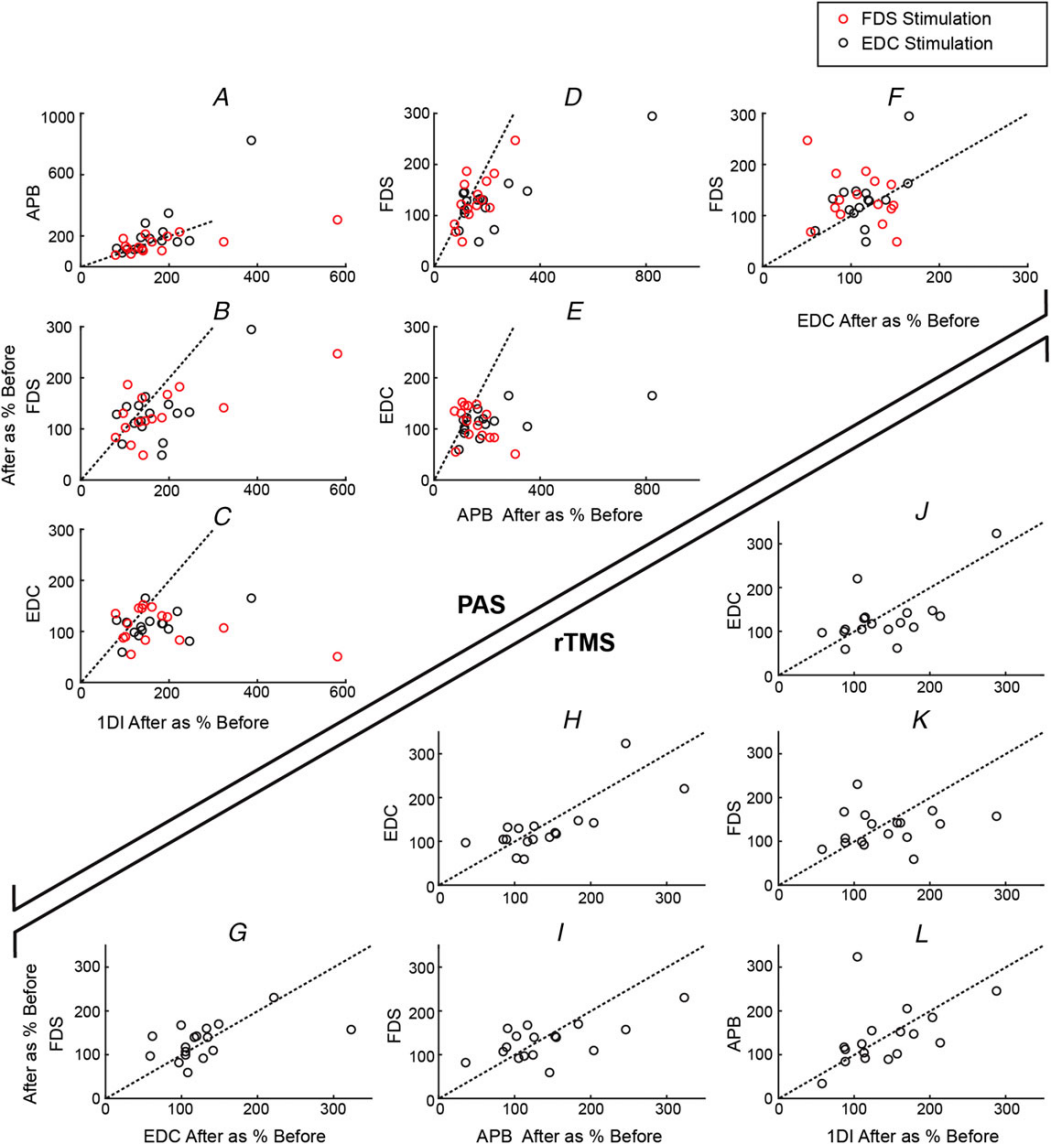
Scatter plots comparing the MEP changes in different muscles across subjects In each case, the MEP size after the intervention is expressed as a percentage of the MEP size before the intervention. All possible pairwise comparisons are shown. *A*–*F*, PAS; red circles indicate studies with FDS stimulation; black circles indicate EDC stimulation. *G*–*L*, rTMS; a dotted line indicates the identity line, corresponding to equal changes in the two muscles plotted. [Color figure can be viewed at wileyonlinelibrary.com]

Figure 3 indicates that, on average, there was a difference in the efficacy of PAS in EDC compared to the other three muscles. Next, we examined how changes in the different muscles inter‐related in a pairwise manner in individual subjects. Figure 4 *A*–*F* presents the changes observed in the different muscles (in each case, the dotted line is the identity line). It is apparent that, when changes in the two intrinsic hand muscles were plotted against each other (Fig. 4 *A*), they tended to lie close to the identity line. By contrast, the equivalent plots for FDS *vs*. either 1DI or APB, (Fig. 4 *B*, *C*) and EDC *vs*. 1DI or APB (Fig. 4 *D*, *E*) showed a tendency for points to lie to the right of the identity line, indicating larger changes in the intrinsic hand muscles than in either forearm muscle. Finally, the plot of changes in FDS *vs*. EDC showed a tendency for larger changes in FDS (Fig. 4 *F*). Although the points in all of these plots have been separated according to whether FDS or EDC stimulation was paired with TMS in the PAS protocol (red and black circles respectively), the site of stimulation appeared to have no influence on the results. These visible trends in the data were confirmed by ANOVA with factors recorded muscle and stimulated muscle, revealing a significant effect of recorded muscle (*P* = 0.0074) but not of stimulated muscle (*P* = 0.37) or their interaction (*P* = 0.37). Subsequent pairwise testing revealed significant differences between the size of changes after PAS between all combinations of muscles (*P* < 0.05), except for 1DI‐APB (paired *t* test).

Figure 4 *D*–*F* shows similar scatter plots comparing the changes induced in different muscles by the rTMS protocol. By contrast to PAS, no consistent differences were observed and ANOVA did not reveal any effect of recorded muscle (*P* = 0.89).

## Discussion

### Use of motor point stimulation to activate peripheral inputs

The original description of PAS by Stefan *et al*. (2000) paired stimulation of the median nerve at the wrist with TMS delivered to the contralateral motor cortex. In the present study, we chose instead to stimulate over the motor point of either FDS or EDC. When working with the intrinsic hand muscles, as described in Stefan et al. (2000), direct nerve stimulation has several advantages. The nerve at the wrist is in a convenient location for stimulation; for the median nerve, only muscles of the thenar eminence are supplied, such that activation will be focussed to an anatomically‐defined muscle group. By contrast, for the forearm flexors or extensors, the relevant nerves (median or radial at the arm) can be relatively deep, such that small movements of the stimulating electrode can generate large changes in efficacy over the long time‐course of a plasticity protocol. Additionally, each nerve innervates multiple muscles (for the median nerve, this includes muscles in both forearm and hand), together with important cutaneous fields. Stimulation of the motor point provides a more stable and better focussed alterative. It is known that such stimulation activates large‐diameter afferent fibres (Bergquist *et al*. 2011) and hence should produce a well‐synchronized afferent volley. Previous studies have used paired motor point stimulation of two muscles to induce a different form of associative plasticity (Ridding & Uy, 2003). In the present study, we show that pairing motor point stimulation with TMS provides a convenient and effective means of delivering PAS.

### Spread of plastic changes

The original description of PAS by Stefan *et al*. (2000) noted that plasticity obeyed the principle of specificity. When median nerve stimulation was paired with TMS, changes were greatest in the median‐innervated APB, small in the ulnar‐innervated abductor digiti minimi (ADM) and not seen at all in distant muscles such as the biceps. Weise *et al*. (2006) reported an even more focal pattern because pairing of the median nerve with TMS in healthy subjects led to changes in APB but not ADM. Plasticity spread to the ADM only in patients with a focal task‐specific dystonia (writer's cramp), which was suggested to be linked to the pathophysiology of this condition. In the present study, we found that stimulation of the motor point of either FDS or EDC induced plastic changes in both forearm flexors and intrinsic hand muscles. This finding probably reflects the highly overlapping representation of muscles controlling the digits in the primary motor cortex (Andersen *et al*. 1975; Poliakov & Schieber, 1999). These muscles are often used in complex and flexible ways to stabilize individual digits, such that simple concepts of antagonist/agonist may be inadequate (Schieber, 1995). In such circumstances, it is perhaps unsurprising that plasticity should not be wholly specific to the stimulated muscle.

### Hierarchy of plastic changes

Irrespective of whether we paired FDS or EDC stimulation with TMS, there was a clear gradient in the magnitude of plastic changes across muscles. Responses were most increased in the intrinsic hand muscles; this observation was all the more striking because these muscles had not been stimulated during the pairing with TMS. Smaller but still significant changes were seen in FDS; again, this was so when either FDS or EDC had been stimulated. Finally, no significant changes were seen in EDC. This suggests that there is a hierarchy in the ability to induce plastic changes in these muscles groups. Importantly, the order revealed in our experiments mirrors that seen after functional recovery, where inputs to flexors and intrinsic hand muscles strengthen, although those to extensors remain weak (Belhaj‐saif & Cheney, 2000; Kamper *et al*. 2003; Zaaimi *et al*. 2012).

Several previous studies have suggested that the cortical control of flexor and extensor muscles differ. In both baboons (Phillips & Porter, 1964) and humans (Palmer & Ashby, 1992), the corticomotoneuronal input to elbow flexors is stronger than to extensors. By contrast, for forearm muscles, there may be a stronger cortical input to extensors (especially to EDC) than to flexors (Clough *et al*. 1968; Cheney & Fetz, 1980), although this bias has not always been observed (Park *et al*. 2004). The cortical area activated using functional imaging is greater for thumb extension than flexion movements (Yue *et al*. 2000). All of these observations suggest fundamental but subtle differences in the cortical control of flexor and extensor muscles.

Two previous studies reported differences in the ability to induce plastic changes in the cortical representation of flexor *vs*. extensor muscles. Vallence *et al*. (2012) recorded the MEPs in forearm muscles before and after a period of deafferentation using ischaemic nerve block. They observed increases in MEPs in flexors but not in extensors during the nerve block, and it was suggested that this reflected differences in the underlying potential for plasticity. Godfrey *et al*. (2013) trained subjects on a finger tracking task, measuring changes in MEPs before and after training. Irrespective of whether tracking was produced against a load that resisted flexion or extension, increases in MEPs were seen for FDS but not for EDC. These results are in good accordance with our own findings. In addition, the results of the present study revealed that intrinsic hand muscles have an even higher potential for plastic change in their cortical representation than the forearm flexors. Krutky and Perreault (2007) measured MEPs before and after a period of training on ballistic movements involving different upper limb joints. A proximodistal gradient was seen, with larger MEP changes induced by training finger than wrist or elbow movements. This may parallel the proximodistal gradient that we observed between intrinsic hand and forearm muscles.

By contrast to the clear differences that we observed in muscle groups following PAS, induction of plasticity using rTMS appeared equally effective for all muscle groups. This may reflect a different site at which synaptic plasticity is induced. PAS depends on an interaction between afferent input and TMS. Following ischaemic nerve block (Vallence *et al*. 2012), afferent input to the cortex is greatly reduced. During training on a demanding motor task (Godfrey *et al*. 2013), proprioceptive and tactile feedback probably play an important role (Todorov & Jordan, 2002; Pipereit *et al*. 2006). A common feature of all studies demonstrating differences between muscle groups may therefore be a dependence on afferent input. Afferent inputs from somatosensory cortex influence superficial cortical layers (Mao *et al*. 2011), in a topographically meaningful way (Rosen & Asanuma, 1972; Cheney & Fetz, 1984). By contrast, the unnatural rTMS stimulus probably influences a wide cortical area across multiple layers. It appears that at least one of the synaptic circuits accessed by rTMS (but not by PAS) can generate equal plasticity in all muscle groups, even the forearm extensors. A previous study also suggested that PAS and rTMS induce plasticity by different underlying mechanisms, with Dileone *et al*. (2016) reporting that patients with a mutation in the HRAS gene (Costello syndrome) exhibited greatly enhanced plastic changes in a PAS protocol compared to controls; by contrast, in these patients, rTMS did not induce plasticity at all. Furthermore, even PAS may act via different mechanisms depending on the time interval between the peripheral stimulus and TMS. Hamada *et al*. (2012, 2014) showed that PAS with a short interstimulus interval (PAS21.5), so that the TMS pulse was delivered just after the arrival of the somatosensory volley (as in the present study), probably involves different interneuronal circuits from PAS with slightly longer intervals (PAS25). The latter can be influenced by modulation of cerebellar excitability (Hamada *et al*. 2012). It remains to be established whether the long‐interval form of PAS and rTMS access distinct or overlapping cortical circuits.

Castel‐Lacanal *et al*. (2007) reported that plastic changes in a forearm extensor muscle could be produced using a modified PAS protocol. In this case, a 500 ms long train of stimuli at 10 Hz was delivered to the extensor carpi radialis motor point prior to the paired TMS stimulus. The long train of afferent stimuli probably generated more diffuse activation than the single shocks used here, possibly paralleling the effects of rTMS in the present study.

PAS is considered to exert its effects predominantly at a cortical level because F waves and the responses to corticospinal tract stimulation at the brainstem remain unchanged after the associative stimulation (Stefan *et al*. 2000). Direct observations of the descending corticospinal volley have also reported increases following the pairing intervention (Di Lazzaro *et al*. 2009). Changes in spinal circuits controlling pre‐synaptic inhibition are also induced (Meunier *et al*. 2007; Lamy *et al*. 2010), although these probbaly do not influence MEPs because corticospinal terminals are not affected by pre‐synaptic inhibition (Nielsen & Petersen, 1994; Jackson *et al*. 2006). Thus, the differences that we observed between muscle groups probably reflect differences in the associated cortical circuitry.

In conclusion, in the present study, we have demonstrated a hierarchy in the potential to induce plastic changes in the cortical output to different muscles controlling the hand. Consistent with previous work, this was only observed in a protocol that relied on integration of sensory and motor inputs. We speculate that this difference between muscle groups may be seen not only in cortical systems, but also in subcortical systems, such as the rubrospinal and reticulospinal tracts, and that the failure to recover extensor function after damage such as stroke could reflect an underlying widespread difference in plasticity. Standard approaches to rehabilitation rely on motor training, in which sensory input is critical. It is possible that exploiting the unnaturally broad access to cortical circuits provided by non‐invasive methods such as rTMS could enhance motor outflow to the extensors in ways not achievable by other means.

## Additional information

### Competing interests

The authors declare that they have no competing interests.

### Author contributions

SNB designed the study. KMRF carried out the experiments and performed the data analysis using scripts written by SNB. Both authors drafted the manuscript and revised it for critical intellectual content. Both authors approved the final version of the manuscript submitted for publication.

### Funding

The present study was supported by the Wellcome Trust (grant number 101002) to SNB.
